# Correlations on the Structure and Properties of Collagen Hydrogels Produced by E-Beam Crosslinking

**DOI:** 10.3390/ma15217663

**Published:** 2022-10-31

**Authors:** Maria Demeter, Ion Călina, Anca Scărișoreanu, Marin Micutz, Mădălina Albu Kaya

**Affiliations:** 1 National Institute for Lasers, Plasma and Radiation Physics (INFLPR), Atomiştilor 409, 077125 Măgurele, Romania; 2 Department of Physical Chemistry, University of Bucharest, 4-12 Regina Elisabeta Blvd., 030018 Bucharest, Romania; 3 Department of Collagen, Division Leather and Footwear Research Institute, National Research and Development Institute for Textiles and Leather (INCDTP), 93 Ion Minulescu Str., 031215 Bucharest, Romania

**Keywords:** collagen, electron beam crosslinking, hydrogel, biomaterials

## Abstract

In this study, a collagen hydrogel using collagen exclusively produced in Romania, was obtained by electron beam (e-beam) crosslinking. The purpose of our study is to obtain new experimental data on the crosslinking of collagen and to predict as faithfully as possible, its behavior at high irradiation doses and energies. To pursue this, the correlations between macromolecular structure and properties of collagen hydrogels were determined by rheological analysis, Fourier Transform Infrared Spectroscopy (FTIR), Scanning Electron Microscopy (SEM), and Differential Scanning Calorimetry (DSC), respectively. The gel fraction, swelling degree, and network parameters of the collagen hydrogels were also investigated at different irradiation doses. Through experimental exploration, we concluded that irradiation with e-beam up to 25 kGy induces crosslinking processes in collagen structure without producing advanced degradation processes. E-beam technology is a great method to develop new materials for medical applications without adding other chemical reagents harmful to human health. The future aim is to develop new wound dressings for rapid healing based on collagen, through irradiation technologies.

## 1. Introduction

Collagen is considered one of the most useful biomaterials for medical applications. It is hydrophilic, exhibits low antigenicity, low inflammatory and cytotoxic responses, good hemostatic properties, and controllable biodegradability. It can be shaped into various forms such as films, discs, sponges, hydrogels, tubes, and powders [1].

Radiation treatments of collagen-based materials started many years ago. Perron and Wright [2] reported that irradiation causes structural alteration in collagen-based materials. Bowes and Moss [3] have established that irradiation with doses from 5 kGy to 500 kGy leads to a loss of crystallinity, an increase in solubility, extensive degradation of molecular structure, and fragmentation into smaller units. The irradiation of collagen solutions up to 0.3 kGy has been shown to result in a reduction in the thermal denaturation temperature of the tropocollagen molecule. Higher radiation doses had little effect on decreasing its denaturation temperature, but the denaturation occurred over a wide temperature range [4]. 

Even at very low irradiation doses of 0.3 kGy, the intermolecular crosslinking of collagen takes place. The crosslinking reaction is visible first of all, through the formation of a soluble gel. On the other hand, at high irradiation doses, collagen becomes completely insoluble [5]. The irradiation of hydrated collagen fibers has been shown to result in a progressive decrease in shrinkage temperature. This parameter shows the integrity of the crystalline structure which depends on the stability of the triple helix from the tropocollagen molecule [6]. Intermolecular crosslinks were produced when the collagen fibers were irradiated in the presence of water. In contrast to this, irradiation in the dry state resulted in the fragmentation of molecules [7]. 

However, its use in the form of hydrogel is still limited. Collagen can be used to improve the biocompatibility of wound dressings, skin substitutes, and scaffolds for tissue engineering. Although collagen has a series of special properties compared to synthetic polymers, frequently, collagen-based hydrogels do not possess adequate mechanical properties. Moreover, the use of collagen for tissue repair is inappropriate due to its low biomechanical stiffness and rapid biodegradation [8,9]. To obtain collagen-based hydrogels with controlled biodegradability, improved biological stability, and good mechanical properties, collagen is generally chemically crosslinked, while physical crosslinking is unusual [10]. The physical methods are preponderantly used for sterilization purposes [11]. Among the physical methods [12,13,14], the least damaging to collagen molecules is e-beam irradiation [15]. E-beam irradiation has an advantage because it does not induce high temperatures, and in a relatively short time various “fast” chemical reactions can take place depending on the applied radiation dose [16]. A series of studies have been carried out on collagen irradiated both in the absence and in the presence of water. It has been found that polypeptide chain scissions predominate when collagen is irradiated in a dry state due to the direct effect of ionizing radiation, and this, in turn, dramatically increases collagen solubility in vitro and the rate of resorption in vivo. A crosslinking reaction of collagen molecules appears in native samples containing water due to the action of highly reactive OH radicals resulting from water radiolysis representing the indirect effect of ionizing radiation [4,17,18]. Riedel et al. irradiated a collagen gel extracted from rat tail and bovine skin using a 10 MeV linear electron accelerator to obtain a new strategy for collagen crosslinking to be used as ECM (extracellular matrix) model systems [19]. 

From the point of view of the studies carried out on collagen gel extracted from calfskin and treated with e-beam to produce a “clean” crosslinking reaction, until now a limited number of publications referring to this subject have been undertaken. Moreover, a detailed study of the treatment of collagen gel with e-beam, using collagen exclusively obtained in Romania, has not been carried out.

For the future development of new hybrid collagen-based biomaterials as wound dressings or tissue regeneration without using potentially toxic chemical reagents, the present study has been undertaken to assess, for the first time, the behavior of collagen gels prepared exclusively by e-beam crosslinking. 

In this context, the purpose of our study is to obtain new experimental data on the e-beam crosslinking of collagen-based hydrogels produced in the local Romanian market, using techniques such as FTIR, DSC, SEM, and rheology coupled with theoretical network models to predict, as faithfully as possible, the behavior of the collagen gel at high irradiation doses and energies.

The newly obtained data will form the basis for the formulation and optimization of the collagen hydrogel using e-beam crosslinking as the main method of manufacturing. Thus, not including chemical crosslinking agents in the recipes for obtaining hydrogel-based collagen, presents the advantage of obtaining biocompatible and biodegradable medical devices.

## 2. Materials and Methods

### 2.1. Materials

Type I acidic collagen gel with a concentration of 2.6% (*w*/*w*) was extracted from calf hide by the currently used technology in the National Research and Development Institute for Textiles and Leather, Collagen Department, Romania [20]. Before e-beam experiments, the collagen gel has been diluted to obtain a collagen aqueous solution with a concentration of 1% (*w*/*w*).

### 2.2. Synthesis of Hydrogels

Collagen gels were weighted exactly (10 g) and placed in hermetically sealed cylindrical containers to prevent air from entering the material. The samples were irradiated at room temperature and atmospheric pressure using the 5 MeV electron accelerator facility (ALID—7) of the Electron Accelerators Laboratory, INFLPR, Măgurele, Romania. All samples were irradiated with doses of 5, 10, 25, 50, and 100 kGy at a dose rate of 4 kGy/min. The nominal dose rate, the absorbed dose, and the e-beam energy (using a standard aluminum wedge) were measured using calibrated graphite calorimeters and B3 radiochromic dosimeter films against standard alanine dosimeters.

### 2.3. Evaluation of Hydrogel Properties

#### 2.3.1. Determination of Gel Fraction

After e-beam irradiation, the hydrogels were dried in an oven at 30 °C (to avoid collagen thermal degradation) for 72 h until a constant weight (W_i_). They were then extracted using deionized (DI) water at room temperature for 48 h and further dried at 30 °C for 72 h (W_d_) until a constant weight. Three replicates of each sample were tested. Finally, the gel fraction (GF) was calculated using the following equation [21]:

(1) $$\mathrm{GF}\left(\%\right)=\left(\frac{W_{d}}{W_{i}}\right)$$

#### 2.3.2. Swelling Degree

The dried hydrogels ($W_{d}$) were immersed in PBS (pH = 7.4) at 37 °C and the swollen samples ($W_{s}$) were removed from the buffer solution at specified time intervals and weighed accurately after removing excess surface water with filter paper. All the measurements for the swelling experiments were performed in triplicate.

The swelling degree (SD) was calculated using the following equation [22]:

(2) $$\mathrm{SD}\left(\%\right)=\frac{\left(W_{s}-W_{d}\right)}{W_{d}}\cdot 100$$

Phosphate buffered saline (PBS, pH = 7.4) was prepared from 8 g NaCl, 0.2 g KCl, 1.44 g Na_2_PO_4_, and 0.24 g KH_2_PO_4_ in 1000 mL deionized water.

#### 2.3.3. Crosslinking Density 

The network parameters as the molecular weight between two successive crosslinking points (M_C_), crosslinking density (Ve), and mesh size (ξ) were calculated using the elastic modulus (G′) values determined in the rheological experiments based on the rubber elasticity theory using the following equations [23,24]:

(3) Mc=AρRT(ν2,r)23(ν2,s)13G′

(4) ξ=ν2,s−13·[Cn(2McMr)]−12·l

(5) $$V_{e}=\frac{\rho }{M_{c}}$$

(6) $$\nu_{2,r\left(s\right)}=\frac{{\left[1+\left(m_{2r\left(s\right)}-1\right)\cdot \rho_{\mathrm{hydrogel}}\right]}^{-1}}{\rho_{\mathrm{solvent}}}$$

where ρ (g/cm^3^) is the polymer density, R is the universal gas constant (8.314 m^3^ Pa/mol·K), T is the absolute experimental temperature (298.15 °K), $\nu_{2,r}$ is the polymer volume fraction after e-beam crosslinking, $\nu_{2,s}$ is the polymer volume fraction of the crosslinked hydrogel in the swollen state, l is the carbon-carbon bond length (0.154 nm), C_n_ is the characteristic ratio (Collagen = 9) [25], M_r_ is the collagen monomeric unit (M_r_ = 321.32 g/mol) and the factor A equals 1 for an affine network and 1–2/ϕ for a phantom network [23]. The polymer volume fractions $\nu_{2,r}$ and $\nu_{2,s}$ were determined using Equation (6).

### 2.4. Instrumental Analysis

#### 2.4.1. Rheological Analysis

The rheological tests were performed using a Micro Fourier Rheometer MFR 2100 (GBC, Australia). Rheological analysis was carried out to determine the magnitude of the storage (G′), loss (G″), complex moduli (G*), and damping (tan δ) to evaluate the viscoelastic properties of collagen hydrogels. The collagen hydrogels of cylindrical shape with an approximate volume of 1 cm^3^ were subjected to the rheological tests using an appropriate deformation and force such as to determine the linear viscoelastic region (LVR), where the storage modulus (G′) is independent of the strain amplitude.

The operating parameters of the instrument during the rheological investigation were as follows: the gap between plates—400 μm, displacement amplitude—0.03 μm, frequency domain—0.05–20.00 Hz (with a step of 0.05 Hz which leads to angular frequencies (ω, in rad/s) of 2π times higher than the corresponding frequencies taken in Hz), equilibration time for each of the isothermal measurements—20 min, 30 scans per one rheogram. The rheograms were acquired considering the same angular frequency range of 1.57–100 rad/s. All the measurements were carried out in triplicate with a relative standard deviation of less than 15% at the same constant temperature of 23.0 ± 0.1 °C.

#### 2.4.2. FTIR Analysis

The FTIR spectra of dried unirradiated and irradiated samples were collected with a Spectrum 100 instrument (Perkin Elmer, Waltham, MA, USA) equipped with a diamond crystal. Spectra were acquired in ATR mode and each spectrum consisted of 30 scans per sample, in the 4000–600 cm^−1^ wavenumber range and a resolution of 4 cm^−1^.

#### 2.4.3. SEM Analysis

The morphology of the dried collagen hydrogels was examined using an SEM (FEI Inspect S model), working at a 20 kV operating voltage. The cross-section of dried hydrogel samples was observed after gold coating. The SEM images were taken at magnifications of 250×, and 500×, respectively.

#### 2.4.4. Thermal Analysis

DSC measurements were performed with the Perkin Elmer Diamond DSC calorimeter under a heating rate of 10 °C/min. The temperature range used for collagen gel samples was 5–140 °C.

## 3. Results and Discussion

### 3.1. Gel Fraction and Swelling Degree

**Figure 1:**
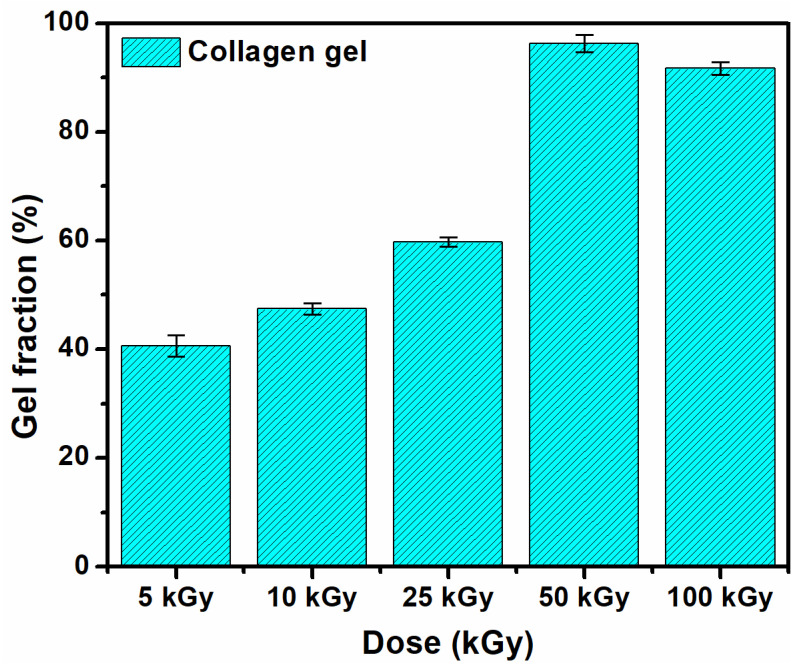
Gel fraction of e-beam crosslinked collagen hydrogels.

The gel fraction of the collagen hydrogels changed significantly depending on the irradiation dose, as shown in Figure 1. The gel fraction percentage increased progressively from 34.9 to 96.3 with the absorbed dose, with a minor decrease at 100 kGy. The content of the gel fraction is almost doubled at 50 kGy by comparison with the gel fraction value at 25 kGy.

One of the most important characteristics of biomedical materials is the swelling of hydrogels in aqueous media similar to biological tissue, as well as their stability in such environments [26].

**Figure 2:**
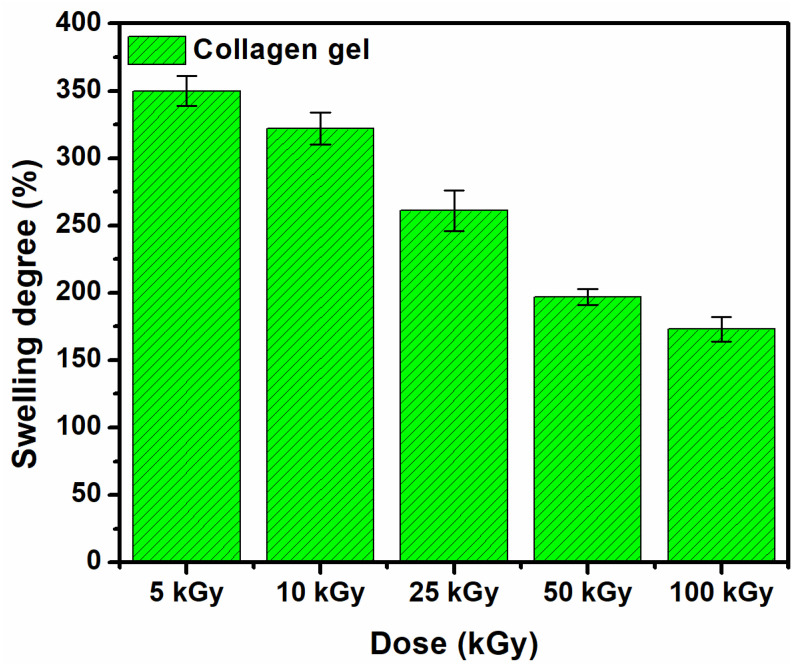
The swelling degree of e-beam crosslinked collagen hydrogels in PBS at 37 °C for 48 h.

Figure 2 shows the swelling degree of the e-beam crosslinked collagen hydrogels in PBS solution at 37 °C. The crosslinked collagen hydrogels show good swelling properties. Swelling normally decreased with dose due to higher crosslinking density at higher doses leading to a decrease in the solvent absorption capacity of the hydrogel. At the sterilization dose, the swelling capacity exceeds 250%, with this value being higher compared to other studies that report data on collagen irradiation [4,15]. The swelling decrease and gel fraction increase with the absorbed dose suggests crosslinking arrangements within collagen macromolecular chains, consequently hydrogel formation [27].

### 3.2. Crosslinking Density

To tailor the ξ, M_C_, and Ve of an e-beam crosslinked hydrogel to be used as a dressing, the most important role is played by the action of radiation. In this regard, the effect of the absorbed dose on the M_C_, Ve, and ξ of hydrogel network parameters was evaluated and the results are shown in Table 1.

The M_C_ varied between 107,538 g/mol and 16,693 g/mol and was dependent on the absorbed dose. Interestingly, the M_C_ of the collagen sample crosslinked with 10 kGy increased significantly even though there were no registered significant differences between 5 kGy and 10 kGy in the G′ modulus.

When the absorbed dose is more than double from 10 to 25 kGy, the radiation effect is almost directly proportional to the G′ modulus and the crosslinking density. Consequently, the effect on the M_C_ parameter is inversely proportional. Up to 50 kGy, we observe moderate changes in terms of the G′ magnitude, as well as in their other parameters. A dose of 100 kGy induced a 20-fold increase in G′ modulus which suggests a strong stiffness of the hydrogels network coupled with the drastic narrowing of their mesh size perhaps unbeneficial to wound dressing purposes. The above findings are well-supported by the swelling experiments which demonstrated the formation of a compact network that restricts the movement of collagen chains and prevents water from entering it.

### 3.3. Rheological Analysis

**Figure 3:**
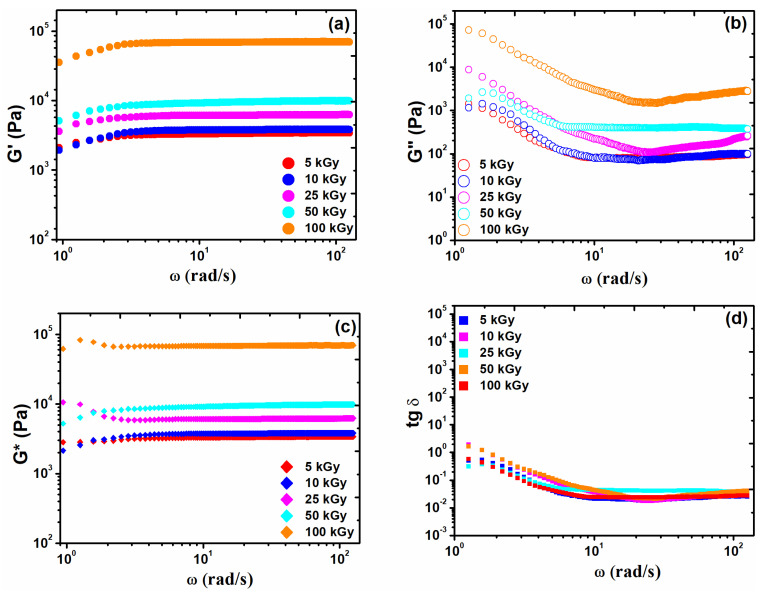
The variation of G′ (**a**), G″ (**b**), G* (**c**), and tan δ (**d**) on the angular frequency (ω) and the absorbed dose, for the collagen hydrogel crosslinked.

The rheology resulting curves are shown in Figure 3. It has been observed that the e-beam crosslinking of the collagen gels leads to a strong increase in G′ modulus up to 69.3 kPa at 100 kGy. The collagen hydrogels have shown an elastic and permanent network structure over the entire range of angular frequencies employed, since G′ > G″. The elastic behavior of the hydrogel is related to the number of crosslinks present. For instance, when G′ becomes larger, this is due to increasing the number of double bonds within the hydrogel network, making it more resistant to external forces [28]. 

Even at a lower irradiation dose, the magnitude of G′ modulus exceeds 3 kPa showing a strong effect on e-beam irradiation. The much higher G′ magnitude at a lower radiation dose could well be associated with the collagen molecular weight. This observation is supported by earlier literature reports which demonstrate that materials with higher molecular weight have more entanglements, resulting in a structure with higher mechanical strength [29]. 

A comparative analysis of G* and G′ moduli is another approach to assess some rheological properties of the irradiated hydrogels. Ideal solids should have G* = G′ and for an ideal liquid G* = G″. Thus, G* may be considered as a measure of the material rigidity, giving the total strength of the investigated hydrogels to an effective strain applied [30]. The frequency dependencies of G′, G″, and G* exhibiting the strong gel character of irradiated collagen gels by comparison with those irradiated at lower doses can be partially replotted into a much more concise and suggestive manner by considering the quantity loss tangent (tan δ = G″/G′). Accordingly, the values of this dimensionless ratio were well below unity for the irradiated mixtures The very small loss tangent values (roughly, more or less, lower than 10^−1^) are indicative of the predominant gel behavior of the irradiated collagen gels [31].

### 3.4. FTIR Analysis

To evaluate the changes produced by e-beam crosslinking in the structure of collagen as well as the preservation of its native structure, the FTIR spectra of unirradiated and irradiated collagen dry films were compared. The FTIR spectra of the native collagen show bands to its specific molecular organization related to the peptide linkages of collagen, namely NH and OH stretching, as amide A (3300–3330 cm^−1^), amide B (3080 cm^−1^), as well as amide I (1650 cm^−1^), amide II and III (1554 and 1240 cm^−1^) [32].

The amide I intensity is a useful tool to evaluate the conformation of collagen molecules in the native and denatured states [33]. Moreover, the amide I band is useful for the identification of the secondary structure of proteins. The amide I band may be assigned to the vibrations of amide carbonyl along the carbonyl backbone. 

Amide II consists of amide NH bending vibrations and CN stretching vibrations [34]. These bands can be used to appreciate the preservation of the triple helical conformation of collagen molecules in a given material [35]. The ratio of amide III absorbance (A_III_), to that from 1450 cm^−1^ (A_1450_), serves as an indicator of the integrity of the triple helical conformation of collagen. The results equal to or higher than unity show its preservation. At the same time, higher differences than 100 cm^−1^ between the frequencies of amide I and amide II, indicate the degradation of collagen [36]. The decrease in amide III band intensity from 1240 cm^−1^ also shows the degradation of the collagen molecule. The amide I/amide A ratio is correlated with the crosslinking degree [37]. 

**Figure 4:**
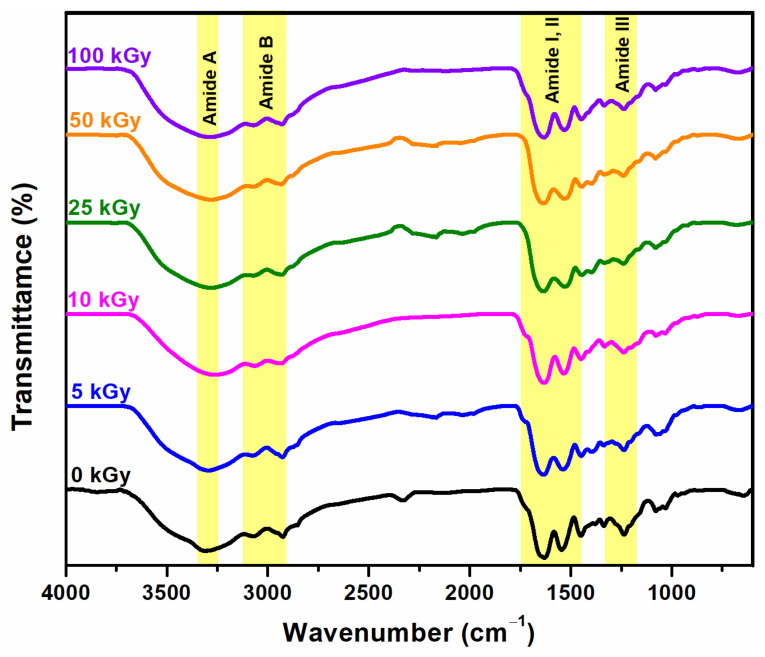
FTIR spectra for uncrosslinked and crosslinked dried collagen hydrogel.

Figure 4 shows the characteristic FT-IR bands of unirradiated and irradiated collagen gels. The amide A and B intensity increased significantly with the increase of the irradiation dose. Moreover, visible changes in the collagen gel consistency were observed. 

Even at the lower dose of 5 kGy, the consistency of the gel became more compact, with a plasticized appearance, without the gel flowing. With the dose increasing, water separation was observed, due to the crosslinking. The processes induced by radiation crosslinking occur by increasing the number of intermolecular bonds between molecular chains of collagen, as existing water molecules cannot be retained within the newly formed structure and are being removed. 

At 0 kGy, the amide A was identified at 3287 cm^−1^, at 5 kGy was shifted to 3301 cm^−1^, and, respectively, to 3270 cm^−1^ at 100 kGy. Amide B has remained unchanged. The band at 2956 cm^−1^ was shifted to 2937 cm^−1^. 

The amide I/Amide A ratio reveals the crosslinking degree and is summarized in Table 2. A higher degree was observed for collagen gel irradiated at 10 kGy, also at this dose other parameters were maintained at a normal level which demonstrates that the collagen molecule was not affected by e-beam crosslinking. Up to 25 kGy, no denaturing effects occur. The amide III/A_1450_ ratio decrease monotonously. The ratio of amide III/1451 cm^−1^ is higher than one unit, showing that collagen triple helix integrity is maintained after e-beam irradiation. Starting with the irradiation dose of 50 kGy, the first denaturation effects on collagen molecules appear. The ratio that reveals the integrity of the collagen triple helix structure has decreased slightly to 0.98. The difference between the absorption bands of amide I and amide II increased with the dose (Table 2). Only at 100 kGy, this ratio was slightly higher than 100 cm^−1^. Since the FT-IR spectra did not show a significant shifting of the characteristic band, it can be concluded that after e-beam crosslinking there were no triple helix-random coil changes.

### 3.5. Surface Characterization—SEM Images

**Figure 5:**
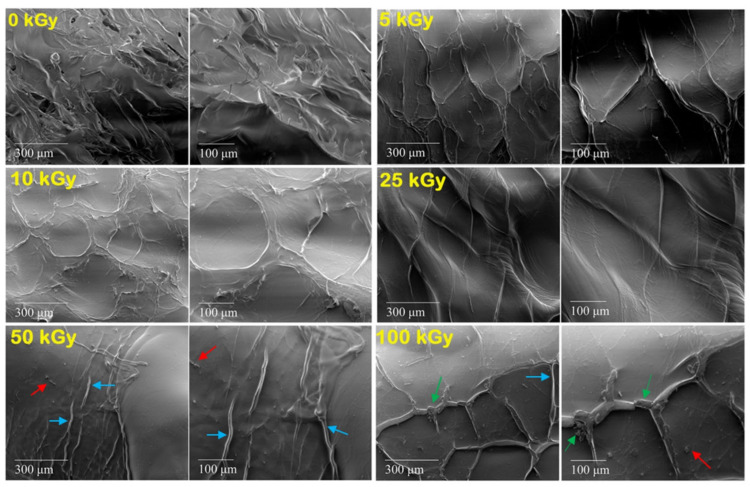
SEM images of non-crosslinked and crosslinked collagen hydrogels. The prominent striations, small fragments of collagen fibers, and fine cracks are marked with arrows.

To emphasize the effect of e-beam crosslinking on collagen surface morphology, scanning electron microscopy was used. To avoid additional mechanical deformation of the collagen hydrogel network usually induced during freeze-drying processes, which is the general procedure used for SEM investigations, we prepared the hydrogel samples by slow drying and constant temperature. By doing so, thermal degradation of the collagen does not occur. This approach shows adequate morphological resolution at the microstructure level where no deformations or collapse of the collagen structure are present. As is shown in Figure 5, the surface morphologies of the 0 kGy sample and e-beam crosslinked collagen hydrogels were vastly different from each other. For example, in the non-crosslinked sample, an irregular but textured structure is observed compared to the crosslinked ones. After irradiation, a compact surface with irregularly arranged fine striations, without pores is observed. Starting with the irradiation dose of 50 kGy, compact structures are also observed, but with many more prominent striations (blue arrows), and small fragments of collagen fibers (red arrows) also present. At 100 kGy, fine cracks in the material are visible (green arrows). These findings prove that the collagen gel became more shrunken. This causes the rapid loss of water from the collagen molecule, increasing the mechanical stiffness of the hydrogel and implicitly its crosslink density. 

According to swelling, rheology, network characterization, and FTIR, above 25 kGy, the effects induced by e-beam action on the collagen gels can be detrimental if used as a component in hydrogel formulation. 

### 3.6. Thermal Analysis

The triple helical structure of the collagen molecule is stabilized by the H bonds formed between the OH and NH_2_ groups of hydroxyproline and glycine. This leads to the formation of a fibrous crystalline zone embedded into an amorphous matrix [38]. Collagen stability can be evaluated by the denaturation temperature (T_d_). The T_d_ expresses the transition temperature of the collagen molecule conformation from triple helix to static coil [39], and can be precisely analyzed by DSC.

The collagen molecule is 10 times more resistant to irradiation than other proteins (e.g., DNA). The most important destructive process observed in its structure under the influence of ionizing radiation (X, e-beams, or γ), is the hydrolysis of peptide bonds. If the irradiation process occurs in solution, destructive processes begin to predominate only at doses exceeding 50 kGy. At suitable doses of irradiation, the main process is crosslinking which takes place through oxidative deamination, forming the C=N or C–C bonds. The newly formed bonds in its structure give it mechanical and thermal resistance. Doses higher than 100 kGy cause the separation of phases and the appearance of a syneretic liquid and leads to the loss of the capabilities to film form, and at the same time, the decrease of solubility [40].

The maximum temperature in the DSC curves reflects the T_d_, and the enthalpy of denaturation (ΔH_d_) gives indications of intra and intermolecular interactions in the sense that if they are inhibited, T_d_ and ΔH_d_ have low values, instead, the pronounced crosslinking leads to increase these values [41].

A series of thermograms presented in the literature for collagen, in the temperature range of 20 to 400 °C highlight two distinct processes [42]. The first endothermic process from 25 to 125 °C is attributed to thermal dehydration, and the second process from 125 to 400 °C is an exothermic event attributed to thermo-oxidative decomposition. 

The peak, symmetry, and area (ΔH_d_) of the peak are the fundamental elements in characterizing the denaturation process of collagen. 

**Figure 6:**
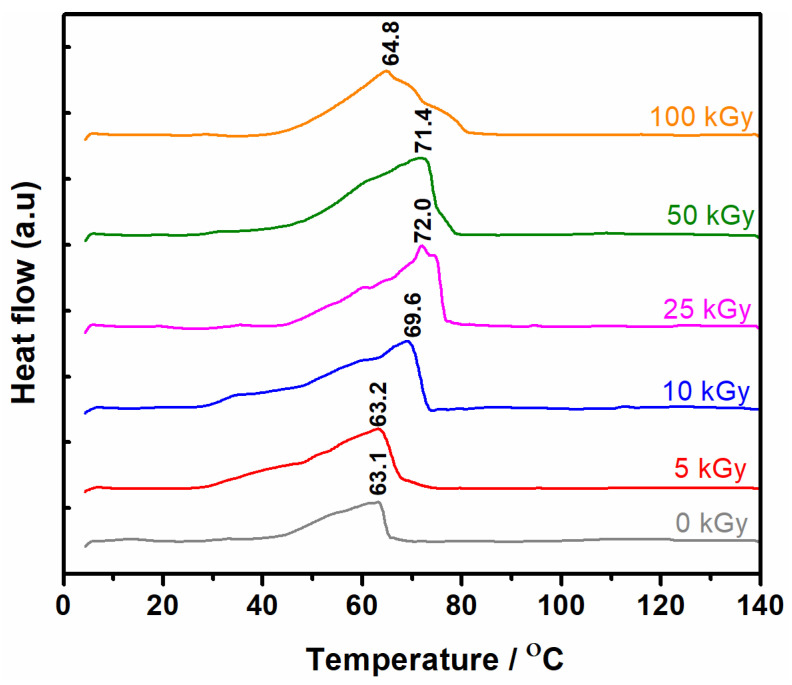
DSC curves for non-crosslinked and crosslinked collagen gels.

Figure 6 shows the DSC thermograms of collagen gels crosslinked with e-beam. In the 40–80 °C range, an endothermic peak with the same start point has been observed for unirradiated and irradiated samples. The T_d_ peak maximum was found between 63.1 °C to 72 °C at 0–25 kGy. Above 25 kGy, at 50 kGy and 100 kGy, respectively, the value of the T_d_ was found between 71.4 °C to 64.8 °C. Therefore, a pronounced decrease in the denaturation temperature is observed with the increase in the irradiation dose. The shifting of endothermic peaks towards higher temperatures can be due to the formation of new chemical bonds, thus high denaturation temperatures are required for their thermal disassembly. 

The damage to collagen molecules can occur through three main processes: gelatinization, thermal destabilization, and denaturation. The enthalpy of denaturation, ΔH_d_ is a direct indication of collagen stability. Its decrease is strongly associated with the progress of degradation [43]. 

As a result, conformational changes occur in the collagen structure, and the decrease in T_d_ is the first indicator of these processes. The conformational changes should exceed a critical level, to produce a full cleavage of the molecule. It should be noted that until the production of a complete destabilization of the collagen molecule, intermediate products are formed as a result of breaking the peptide bonds without the triple helix structure being affected. These processes are also visible in the DSC analysis through decreases in the T_d_. For the irreversible denaturation of the collagen molecule, a sufficiently large number of covalent bonds should be broken, practically producing a shortening of the peptide chains, resulting in an insufficient number of hydrogen bonds to stabilize the helical structure [44].

**Figure 7:**
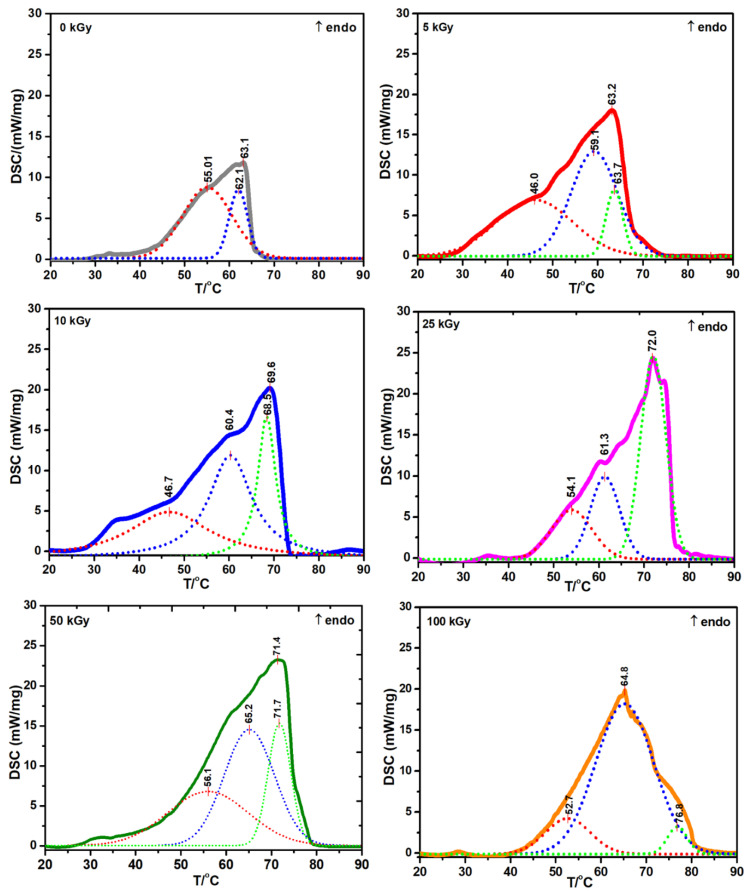
DSC curves for crosslinked collagen hydrogel.

Figure 7 shows the characteristic DSC curves obtained for non-irradiated and irradiated collagen gels. In the temperature range of 20 to 80 °C, a wide endothermic peak, with multiple inflections appears. From the DSC data obtained it is found that with the progressive heating of collagen gels, denaturation is characterized by an endothermic process.

The T_d_ was considered the temperature corresponding to the peak maximum, and ΔH_d_ was calculated by integrating each DSC curve, which was then related to the mass of the sample to be analyzed. For an accurate calculation of the enthalpy and to improve the symmetry of the peaks, the experimental DSC data were processed using the “Multiple Peak Analysis” function of the OriginPro program.

Table 3 shows that for the non-irradiated collagen sample, the temperature corresponding to a better separated endothermic peak was found at 63.1 °C and two endothermic peaks were identified at 55.0 and 62.1 °C, following deconvolutions. 

For the irradiated ones with 5 kGy, the peak maximum is located at 63.2 °C, and after deconvolution, there are three subsequent endothermic peaks at 46 °C, 59.1 °C, and 63.7 °C. The temperature of 46 °C can be attributed to the gelatinization process of collagen.

As the irradiation dose increased to 25 kGy, an increase in the T_d_ and the corresponding ΔH_d_ was observed. The T_1_ temperature associated with the gelatinization process increases with the dose. The T_2_ and T_3_ peaks were linked with the thermal destabilization and denaturation processes. In the case of the thermal destabilization process (peak T_2_), the maximum required temperature was 65.3 °C. The denaturation process (peak T_3_) needed a temperature of 76.8 °C. Decomposition enthalpy is observed to increase with a dose of up to 25 kGy. At 100 kGy, the ΔH_d_ decreased drastically up to 92.1 J·g^−1^. 

According to some authors, the modified collagen shows a T_d_ between 69.0 °C to 84.4 °C, unmodified collagen shows a T_d_ at 52.2 °C, and gelatinized collagen presents a T_d_ of 42.8 °C [44]. The T_d_ describes the hydrothermal stability of the collagen. The higher the T_d_, the higher the collagen stability. The crosslinking increases T_d_ as a consequence of dehydration produced by the e-beam irradiation. The opposite effect, such as hydrolysis of peptide chains, oxidation, or destabilizing of the triple helix is visible by a T_d_ decrease [45]. 

Considering data from the literature, and comparing our data, the e-beam crosslinking of collagen gels produces a decrease of T_d_ and ΔH_d_ parameters only at irradiation doses of 50, and 100 kGy, respectively. 

Moreover, the enthalpy of collagen denaturation was found to be around ΔH_d_ = 31.0 J·g^−1^ [46]. The ΔH_d_ above 25 kGy decreased to 19.8 J·g^−1^, and to 4.18 J·g^−1^, respectively, suggesting a considerable decrease in the hydration level. If the hydration level is lower, the collagen triple helix structure is maintained only by a relatively small number of hydrogen bonds, which could produce an irreversible change in its structure [47]. The thermal analysis data are in good agreement with the swelling, rheology, and network parameters, in the sense that through all three methods, the crosslinking and degradation process were highlighted. 

## 4. Conclusions

In this study, we obtained the optimum preparation conditions of collagen hydrogel by e-beam crosslinking. To determine the hydrogel properties, the correlations between the macroscopic structure and the network properties were studied. 

The gel fraction and swelling experiments showed the formation of a crosslinked and stable collagen hydrogel according to the radiation dose. 

The molecular weight between the two crosslink points decreased seven-fold at 100 kGy showing the formation of a strong hydrogel network. However, such a high radiation dose is detrimental to the crosslinking process. 

The rheological measurements confirmed the elastic character of collagen hydrogels and indicate that the high molecular weight of collagen and higher radiation dose led to obtaining hydrogels with a high degree of crosslinking. The rheological tests showed increases in elastic modulus over two orders of magnitude up to 50 kGy and seven times higher at 100 kGy. Such effects have been associated with an increase in crosslink density and a strong shrinking of collagen molecules at 100 kGy.

The results of FTIR analysis show no significant changes after e-beam irradiation up to 25 kGy.

The SEM images showed a transition from an irregularly textured structure characteristic of the non-irradiated gels to a more compact structure with prominent striations for the irradiated ones. At 100 kGy, fine cracks in the material are visible due to the advanced shrinkage of the collagen gel. This causes the rapid loss of water from the collagen molecule, increasing the mechanical stiffness of the hydrogel up to 20-fold, which is unbeneficial for wound dressing purposes. 

The DSC data suggests that with increasing denaturation temperatures up to 25 kGy, new chemical bonds produced by e-beam irradiation were formed. In contrast, decreasing denaturation temperature and enthalpy after 50 kGy, and 100 kGy, respectively, have been associated with the loss of the triple-helix structure of the collagen molecule.

These new data will be consistent for future development of collagen-based-hydrogel formulations as dressings for skin regeneration using the main method of processing which is e-beam crosslinking.

Furthermore, experiments will be continued with the inclusion of collagen in mixtures with other natural polymers to obtain new hydrogels produced by e-beam crosslinking to develop new materials that can be used as medical devices for wound healing.

## Figures and Tables

**Table 1:** The effect of absorbed dose on network parameters of collagen hydrogels.

Dose	ρ	G′	G″	Mc	Ve × 10^−5^	ξ
(kGy)	(g/cm^3^)	(Pa)	(Pa)	(g/mol)	(mol/cm^3^)	(nm)
5	1.0011	3386	86	107,538	0.93	23.06
10	1.0105	3807	89	145,522	0.69	23.67
25	1.0087	6174	174	99,356	1.02	18.62
50	1.0010	9713	395	95,903	1.04	16.93
100	1.0211	69,315	2267	16,693	6.11	6.61

**Table 2:** FTIR characteristics of e-beam crosslinked collagen hydrogel at different doses.

Dose, (kGy)	Amide I/Amide A	Amide III/A_1450_	Δυ = υ_I_ − υ_II_ (cm^−1^)
5	2.70	1.29	84.38
10	3.57	1.18	87.86
25	2.50	1.04	94.98
50	2.83	0.98	96.23
100	1.92	0.82	101.07

**Table 3:** Thermodynamic parameters acquired using Multiple Peak Analysis of DSC data for e-beam crosslinked collagen hydrogel.

Dose	T_d_	ΔH_d_	T_1_	ΔH_1_	T_2_	ΔH_2_	T_3_	ΔH_3_
(kGy)	(°C)	(J·g^−1^)	(°C)	(J·g^−1^)	(°C)	(J·g^−1^)	(°C)	(J·g^−1^)
0	63.1	90.9	-	-	55.0	26.6	62.1	9.51
5	63.2	92.2	46.0	31.5	59.1	35.8	63.7	8.15
10	69.6	152.7	46.7	36.0	60.4	38.6	68.5	19.6
25	72.0	155.3	54.1	13.9	61.3	16.8	71.9	35.9
50	71.4	146. 6	56.1	31.1	65.2	41.7	71.7	19.8
100	64.8	92.1	52.7	12.36	65.3	68.2	76.8	4.18

## Data Availability

Not applicable.
